# Persistent SARS-CoV-2 Positive Tests in Neonates: Clinical Outcomes, Transmission Pathways, and Immune Vulnerability—Case Series

**DOI:** 10.3390/children13020264

**Published:** 2026-02-13

**Authors:** Orly Grobeisen-Duque, Oscar Villavicencio-Carrisoza, Mariana Diaz-Garcia, Monica Selena Fonseca-Perez, Miguel Angel Diaz-Zurita, Moises Leon-Juarez, Martha Lucia Granados-Cepeda, Victor Hugo Ramirez-Santes, Maria Isabel Villegas-Mota, Mario Rodriguez-Bosch, Rene Humberto Barrera-Reyes, Irma Alejandra Coronado-Zarco, Sandra Acevedo-Gallegos, Carolina Valencia-Contreras, Manuel Cortes-Bonilla, Jorge Arturo Cardona-Perez, Addy Cecilia Helguera-Repetto

**Affiliations:** 1 Departamento de Inmunobioquímica, Instituto Nacional de Perinatología Isidro Espinosa de los Reyes, Ciudad de México 11000, Mexico; orly.grobeisen@gmail.com (G.-D.O.); mariana.diaz@iest.edu.mx (D.-G.M.); mselena.fp@gmail.com (F.-P.M.S.); mikedizuri@gmail.com (D.-Z.M.A.); moisesleoninper@gmail.com (L.-J.M.); 2 Departamento de Medicina traslacional, Instituto Nacional de Perinatología Isidro Espinosa de los Reyes, Ciudad de México 11000, Mexico; cuauqbp@gmail.com; 3 Escuela Nacional de Ciencias Biológicas, Instituto Politécnico Nacional, Ciudad de México 11340, Mexico; 4 Dirección Médica, Instituto Nacional de Perinatología Isidro Espinosa de los Reyes, Ciudad de México 11000, Mexico; marthagranadoscepeda@gmail.com (G.-C.M.L.); manuelcortesb@yahoo.com.mx (C.-B.M.); 5 Departamento de Obstetricia, Instituto Nacional de Perinatología Isidro Espinosa de los Reyes, Ciudad de México 11000, Mexico; vhrsan@hotmail.com; 6 Departamento de Epidemiología Hospitalaria, Instituto Nacional de Perinatología Isidro Espinosa de los Reyes, Ciudad de México 11000, Mexico; isabelvillegasmota@gmail.com; 7 Subdirección de Ginecología y Obstetricia, Instituto Nacional de Perinatología Isidro Espinosa de los Reyes, Ciudad de México 11000, Mexico; marrodbosch@yahoo.com; 8 Coordinación de Cuidados Inmediatos al Recién Nacido, Instituto Nacional de Perinatología Isidro Espinosa de los Reyes, Ciudad de México 11000, Mexico; rene7bar@yahoo.es (B.-R.R.H.); ucincmn20@hotmail.com (C.-Z.I.A.); 9 Departamento de Medicina Fetal, Instituto Nacional de Perinatología Isidro Espinosa de los Reyes, Ciudad de México 11000, Mexico; dracevedo_sandra@yahoo.com.mx; 10 Subdirección de Neonatología, Instituto Nacional de Perinatología Isidro Espinosa de los Reyes, Ciudad de México 11000, Mexico; dra.cvalencia@gmail.com; 11 Dirección General, Instituto Nacional de Perinatología Isidro Espinosa de los Reyes, Ciudad de México 11000, Mexico; acardonadr@gmail.com

**Keywords:** case report, COVID-19, SARS-CoV-2, neonatal persistence, neonatal infection, superinfection, neonatal death

## Abstract

**Highlights:**

**What are the main findings?**

- Neonates with persistent SARS-CoV-2 positivity showed high rates of adverse outcomes, including sepsis, superinfections, inadequate weight gain, and mortality, particularly among preterm and low-birth-weight infants.
- Persistent viral detection during hospitalization was associated with prolonged inflammatory states and increased susceptibility to secondary bacterial and fungal infections.

**What are the implications of the main findings?**

- Persistent SARS-CoV-2 positivity represents a significant clinical risk in neonates, underscoring the need for close monitoring, early identification of inflammation, and prevention of superinfections.

**Abstract:**

**Background**: In 2020, the World Health Organization declared a Public Health Emergency of International Concern due to the global outbreak of SARS-CoV-2. Recognized as a severe and highly contagious disease, it affected both the adult and pediatric population. However, due to the early timing of the pandemic, limited research was conducted in the perinatal field, leaving many questions regarding the true impact of maternal transmission to fetuses and its consequences during the neonatal period. **Methods**: In this case series, we reviewed data from ten newborns delivered in the Instituto Nacional de Perinatología (INPer) in Mexico City (tertiary referral institute), all from high-risk pregnancies, between November 2020 and January 2021, all of whom tested positive for SARS-CoV-2 at various points during their hospital stay. **Results**: Despite showing correct extrauterine adaptation after birth, several of them developed complications such as sepsis, superinfections, inadequate weight gain, and, in some cases, death. **Conclusions**: These results highlight the urgent need for targeted neonatal care protocols and further research to better understand the impact of persistent viral positivity and immune vulnerability in this population.

## 1. Introduction

On 30 January 2020, the World Health Organization (WHO) declared a Public Health Emergency of International Concern (PHEIC) due to the global outbreak of the infectious disease caused by Severe Acute Respiratory Syndrome Coronavirus 2 (SARS-CoV-2) [1]. Over the past five years, healthcare professionals have continuously analyzed the microbiological, epidemiological, and clinical characteristics of this virus to improve understanding, prevention, diagnosis, and treatment.

To date, SARS-CoV-2 and its variants have been recognized as highly contagious, generally causing more severe illness in adults than children [2]. However, data on pediatric cases remain contradictory. Although infections are less common in children, a pattern has emerged, suggesting that younger patients tend to experience more severe clinical courses, with neonates being particularly vulnerable. Studies have shown that most infected children are asymptomatic or present with mild to moderate symptoms. Nevertheless, neonates are at a higher risk of developing severe infections, even though most cases do not meet the criteria for acute respiratory distress syndrome (ARDS) [2,3,4].

During the SARS-CoV-2 pandemic, research primarily focused on the effects of the virus in adults and children, while less attention was given to its impact on neonates with perinatal exposure. SARS-CoV-2 infection in newborns can lead to a wide spectrum of outcomes, ranging from mild respiratory symptoms such as cough, rhinorrhea, and fever to more severe conditions like lethargy, poor feeding, neonatal sepsis, and even neonatal death [5]. The exact mode of SARS-CoV-2 transmission in neonates remains unclear, and vertical transmission cannot be ruled out [6].

Due to risk factors such as neonatal prematurity, inadequate prenatal care, congenital anomalies, and infection, newborns are particularly vulnerable to adverse outcomes, including neonatal death, within the first 28 days of life [7]. Although neonatal death in newborns infected with SARS-CoV-2 is generally low, additional factors such as preterm birth, often triggered by maternal inflammation, and fetal exposure to pro-inflammatory cytokines produced in response to maternal SARS-CoV-2 infection during placental transmission may create a high-risk environment, potentially increasing neonatal mortality directly or indirectly [8,9,10].

In this retrospective case series, we aim to describe the maternal and neonatal clinical characteristics and outcomes of ten neonates diagnosed with SARS-CoV-2 at the Instituto Nacional de Perinatología Isidro Espinosa de los Reyes (INPer), a tertiary-level hospital in Mexico City. Our goal is to identify potential patterns in neonatal infection that may guide future research and clinical management.

## 2. Case Descriptions

**Figure 1:**
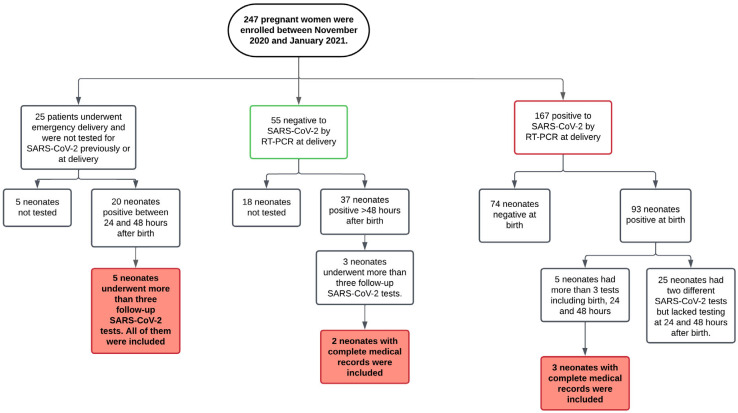
Flowchart of the selection of SARS-CoV-2-positive and persistent neonates. Between November 2020 and January 2021, 247 pregnant women were attended to at the INPer. As part of the universal SARS-CoV-2 screening protocol, all women with scheduled medical follow-up were tested before or at the time of delivery; only those with emergency deliveries were not tested. All neonates born to SARS-CoV-2-positive mothers were tested, while neonates born to negative or untested mothers were evaluated only if they presented clinical symptoms. Thirteen neonates underwent follow-up testing, but complete medical records were available for only ten.

We present ten cases of neonates who tested positive for SARS-CoV-2, with persistent positivity throughout their hospital stay. They were admitted to the Neonatal Intensive Care Unit at the INPer in Mexico City, a tertiary-level national institute specializing in perinatal health. All mothers had received most of their prenatal care at the same hospital, having been identified through institutional criteria for pregnancy follow-up. Cases were included based on SARS-CoV-2 positivity and persistence and only if complete maternal data were available. Data collection was made upon neonates delivered between November 2020 and January 2021. Once a neonate tested positive for SARS-CoV-2, follow-up molecular testing was not mandatory. Consequently, we have only a small cohort of neonates for whom data on the persistence of SARS-CoV-2 RNA are available (Figure 1).

Data for this case series were retrieved from electronic medical records. Due to the SARS-CoV-2 pandemic, almost all mothers were tested for the virus using nasopharyngeal (NP) and oropharyngeal (OP) swabs upon admission. Neonates born to SARS-CoV-2-positive mothers were tested immediately after birth using saliva and rectal samples. Neonates born to negative mothers or unscreened mothers were tested only if they developed symptoms. Unscreened mothers were not primarily tested due to emergency deliveries; however, if their neonates tested positive, or they later presented mild symptoms suggestive of SARS-CoV-2, maternal testing was subsequently performed to assess possible transmission.

All neonates who tested positive for SARS-CoV-2 were transferred to a dedicated isolation area specifically designated for SARS-CoV-2-positive mothers and their newborns, where specialized care protocols were implemented. Access to this area was strictly controlled and limited to trained personnel using the appropriate personal protective equipment (PPE) to prevent cross-contamination. Both mothers and infants underwent regular clinical assessments, laboratory testing, and continuous monitoring to evaluate their overall health and progress. This isolation area was completely separated from the general neonatal care units. Whenever a neonate tested positive for SARS-CoV-2, they were immediately transferred to this unit, and newborns in adjacent hospital bassinets were closely monitored for the development of symptoms.

### 2.1. Specimen Collection and Virus Detection

Pregnant women admitted for labor at INPer underwent a standardized SARS-CoV-2 admission protocol, which included clinical triage and NP/OP swab testing. In emergency deliveries, maternal testing was conducted only if the neonate later tested positive or the mother developed mild symptoms suggestive of SARS-CoV-2.

It is worth noting that the institute exclusively attends to patients presenting with conditions such as obesity, adolescent or advanced maternal age, gestational or preexisting diabetes mellitus, multiple pregnancies, thyroid disorders, uterine myomatosis, cervical insufficiency, hypertension, a history of miscarriage, cardiac disease, fetal anomalies, autoimmune disorders, HIV infection, and placental complications; therefore, the findings may not be representative of the general obstetric population.

RNA was isolated from maternal and neonatal swabs using the Quick-RNA Viral Kit (ZYMO Research, Irvine, CA, USA), following the manufacturer’s instructions. RT-PCR Berlin protocol was followed for SARS-CoV-2 detection [11].

### 2.2. Transmission Classification

We used the WHO classification for neonatal transmission of SARS-CoV-2. According to the WHO, transmission is categorized based on the timing of exposure: in utero, *intrapartum*, or *early postnatal*. Each category can be further subclassified by the certainty of transmission into *confirmed*, *possible*, *unlikely,* and *indeterminate*.

#### 2.2.1. In Utero Transmission

These cases involve mothers with suspected, probable, or confirmed SARS-CoV-2 infection at any point during pregnancy, resulting in potential fetal exposure in utero and evidence of viral persistence or immune response after birth. Confirmation must be established through RT-PCR testing within the first 24–48 h of life or serologic testing (IgM/IgA) between 24 h and <7 days of life.

A *confirmed* classification requires evidence of in utero exposure, such as a positive RT-PCR from a sterile or non-sterile sample, placental tissue, or a positive serology, along with a positive RT-PCR from a sterile sample taken 24–48 h after birth. The *possible* category is similar but allows for a positive RT-PCR from a non-sterile sample or a positive serology alone in the neonate during the first 24–48 h. For *unlikely* cases, although there is evidence of in utero exposure, there is no viral persistence or immune response in the neonate or vice versa. An *indeterminate* classification is used when no tests were performed to assess in utero exposure or when neither viral persistence nor immune response was confirmed in the neonate [12].

#### 2.2.2. Intrapartum Transmission

These cases involve mothers with suspected, probable, or confirmed SARS-CoV-2 infection around the time of delivery, with no confirmed in utero exposure (either negative or untested), and with evidence of viral persistence or immune response acquired during birth.

There are three subcategories. In *confirmed* cases, the absence of in utero transmission must be confirmed by a negative test, and the neonate must have a positive SARS-CoV-2 test within 24– 48 h, either by RT-PCR from a sterile sample or RT-PCR from a non-sterile sample confirmed by a second positive test between 48 h and 10 days or a positive serology between days 7–14, with a second confirmational test within 10 days. In the *possible* classification, the same neonatal criteria apply, but in utero transmission was not ruled out due to a lack of testing. In the *unlikely* category, the neonate has a positive RT-PCR from a sterile or non-sterile sample or a positive serology between days 7–14, but a second test is negative, making the intrapartum transmission less likely [12].

#### 2.2.3. Early Postpartum Transmission

These cases also involve mothers infected around the time of birth, with no confirmed in utero or intrapartum exposure (either negative or untested) and with evidence of infection or immune response in the neonate occurring after 48 h of life.

*Confirmed* early postnatal transmission requires a negative test ruling out in utero and intrapartum transmission and a positive RT-PCR from a sterile sample, a non-sterile sample taken after 48 h, confirmed with a second positive test within 10 days, or a positive serology after day 14 confirmed by a second test within 10 days. In the *possible* category, there is no test to confirm or rule out earlier transmission, but the criteria for early postnatal exposure are met. *Unlikely* classification meets the same exposure criteria as *possible* but includes a negative second test following an initial positive RT-PCR or serology after 14 days. Finally, *indeterminate* cases meet the same criteria as *unlikely*, except the second confirmatory test is not performed after the first positive RT-PCR or serology result, leaving the classification uncertain [12].

### 2.3. Birth Weight Classification and Weight Gain Classification

Birth weight was classified using two complementary approaches. First, according to standard WHO definitions, neonates were categorized by absolute birth weight as follows: extremely low birth weight (ELBW) if weight was below 1000 g, very low weight (VLBW) between 1000 and 1499 g, low birth weight (LBW) if weight was below 2500 g, adequate birth weight (ABW) between 2500 g and 3999 g, and excessive birth weight (EBW) if weight was above 4000 g. Second, to account for gestational age, birth weight was also assessed using Fenton growth charts, which provide gestational age-specific percentiles. Neonates were classified as small for gestational age (SGA) if their birth weight was below the 10th percentile, appropriate for gestational age (AGA) if it was between the 10th and 90th percentiles, and large for gestational age (LGA) if it was above the 90th percentile for gestational age. This dual approach allowed for both an absolute weight classification and a gestational age-adjusted assessment, ensuring consistent categorization across both preterm and term neonates.

General guidelines about weight gain during infancy are primarily established for neonates with an adequate gestational age at birth. Due to the lack of standardization for growth in preterm neonates, we based our analysis on expected weight gain patterns. The typical weight loss of up to 10% in the first days of life, followed by weight recovery between days 10–14 postpartum, was considered in order to make the calculations more accurate [13]. In that sense, we considered the expected physiological pattern of postnatal weight gain during early life. Newborns typically gain approximately 30 g (1 ounce) per day from birth to three months of age, reflecting a rapid growth and high metabolic demand. After this period, the average weight gain decreases to about 20 g (0.67 ounces) per day in older infants, consistent with the gradual stabilization of growth velocity.

Based on these parameters, neonates were classified as having inadequate, adequate, or excessive weight gain depending on whether their weight trajectory fell below, within, or above the expected range, respectively.

### 2.4. Neonate Definition

The term neonate is defined, as per international guidelines, as an infant’s first 28 days of life. At INPer, this definition is extended to include babies who have not been discharged from the hospital since birth regardless of whether they are beyond 28 days of age. In this cases series, we will use the term neonate to refer to infants meeting either of these two criteria [14].

### 2.5. Ethical Approval

This study is part of two research projects (2020-1-31 and 2020-1-32), both approved by the Ethics in Research Committee (ERC) of the INPer. The study was conducted in accordance with the Declaration of Helsinki and followed Strengthening the Reporting of Observational Studies in Epidemiology (STROBE) guidelines (www.equator-network.org, accessed on 17 November 2020). All patients receiving care at INPer provide informed consent and receive a written privacy notice regarding the use of their personal data recorded in electronic medical records.

## 3. Results

### 3.1. Maternal Clinical Characteristics

Among the ten studied neonates, eight were singleton pregnancies, while two were twin gestations (cases 4 and 8; cases 5 and 6). The mothers were referred to INPer from secondary-care hospitals for prenatal follow-up. The maternal age range was between 19 and 37 years old. Six were primiparous, while four were multiparous. Of the multiparous women, one had a previous spontaneous abortion, and the other three had previous live births via cesarean delivery. No relevant gynecological or obstetric history was found that could significantly impact the analysis of this case series (Table 1).

Regarding maternal comorbidities, two mothers were diagnosed with gestational diabetes but none had pregnancy-related hypertensive disorders. No other significant maternal disease was reported, classifying them as generally healthy during pregnancy. All deliveries were performed via cesarean section. Two mothers experienced obstetric hemorrhage, which was promptly managed by the multidisciplinary obstetric teams (Table 1).

Among the eight mothers, three had term deliveries (two early term and one full term) and five had preterm births (including both twin pregnancies). Five of the studied mothers tested positive during the triage via NP and OP swabs and were transferred to a designated isolation area for delivery and postpartum care. None exhibited moderate or severe SARS-CoV-2 symptoms (Table 1).

### 3.2. Neonatal Outcomes and Clinical Course

All ten neonates were delivered via cesarean section. The gestational age range was between 31 and 39.3 weeks. Six were male, and four were female. Due to prematurity, four neonates required fetal pulmonary maturation with dexamethasone 48 h prior to birth. All showed appropriate extrauterine adaptation, with 5 min Apgar scores ranging from 8 to 9. Regarding blood type, nine neonates had an O+ blood type, while one had A+, differing from the predominant pattern (Table 2).

At birth, only two neonates had ABW, both of whom were also AGA. The remaining eight had LBW or VLBW, with the lowest weights observed in the twin neonates born at 31 weeks of gestation, cases 5 and 6. From those with VLBW, one showed to be SGA and one was AGA. Among those six with LBW, three were SGA and three were AGA.

Without exception, all neonates were diagnosed with SARS-CoV-2 during their hospital stage regardless of their mother’s infection status at the time of delivery. Due to their pro-inflammatory and immunologically vulnerable state, all neonates were admitted to the SARS-CoV-2 isolation area, separate from uninfected newborns. They were managed by a multidisciplinary team, including neonatologists, pediatricians, and pneumologists. Regular laboratory monitoring was performed, including D-dimer testing, with elevated levels observed in three of the ten neonates during their hospitalization (Table 2).

During their stay, three neonates developed a co-infection: case 1 and case 4 with a fungal pathogen, *Candida parapsilosis* and *Candida albicans,* respectively, and case 3 with a bacterial infection caused by *Klebsiella pneumoniae*. Treatment was individualized based on clinical presentation and diagnostic findings. None of them were treated at any point with corticosteroids after birth (Table 2). These three cases were SARS-CoV-2-positive prior to the onset of bacterial or fungal infections (more than 1 month after birth). Additionally, all neonates were premature and showed inadequate weight gain during hospitalization. These factors suggest that their immune systems were already under stress, increasing their susceptibility to secondary infections. In this context, SARS-CoV-2 likely acted as a predisposing factor, although the cause of death listed on the death certificate was “septic shock, late sepsis”.

**Figure 2:**
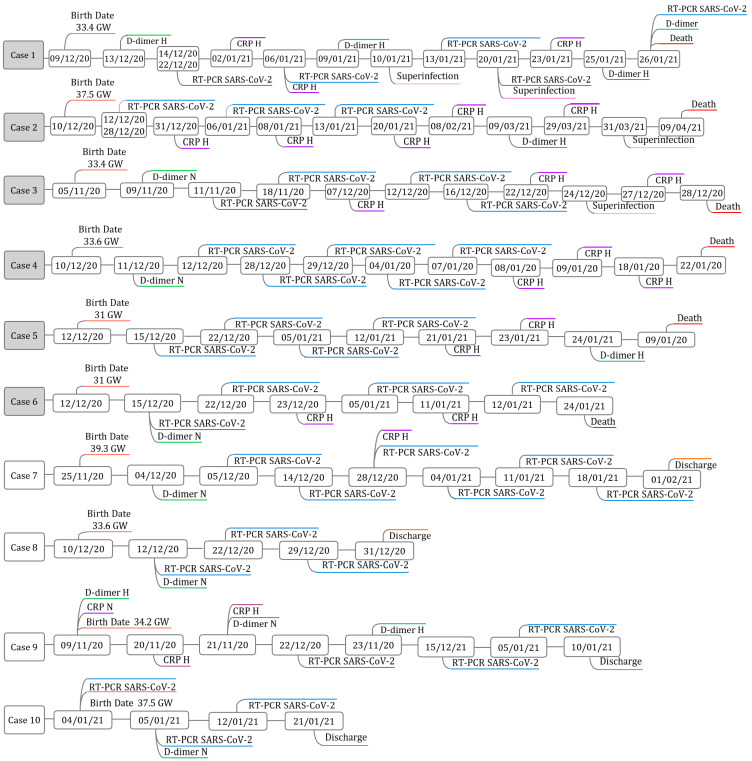
Timeline of findings for each case of SARS-CoV-2 neonatal persistence.

Figure 2 presents the timelines of the ten cases, showing the timing of viral detection, levels of inflammatory markers (CRP and D-dimer) and the points at which superinfections were detected to better illustrate viral initial identification, persistence, and its clinical implications.

Neonatal cases were included if they had clinical and laboratory follow-up with at least three positive SARS-CoV-2 tests on different days during hospitalization. The timeline shows the points of viral detection from birth until discharge and illustrates the timing of inflammatory marker measurements and infection-related complications. CRP and D-dimer results are indicated as N (normal levels) or H (high levels). GW stands for gestational weeks.

Respiratory support was provided as needed. None of the neonates required invasive mechanical ventilation; however, all received some form of non-invasive respiratory support, including oxygen masks (direct or indirect), nasal cannulas, or continuous positive airway pressure (CPAP) (Table 2).

The duration of the hospitalization stay range was between 18 and 118 days. Neonatal weight and length were recorded at the time of medical discharge, whether due to clinical improvement or death. (Table 2).

Of the ten neonates, six died due to different complications observed. Among the four survivors, two experienced excessive weight gain during hospitalization, while two had inadequate weight gain. All six deceased neonates exhibited an inadequate weight gain relative to their gestational age. Among the deceased neonates, five were preterm births, while only two preterm births were recorded among the surviving group. Of the twin pregnancies, one pair (case 5 and 6) resulted in the death of both neonates, while in the second pair (case 4 and case 8), one survived and the other died. The cause of death was attributable to septic shock or sepsis (Table 2).

### 3.3. Timing of Infection and Transmission Classification

Eight of the ten neonates tested positive on their first test. The remaining two, case 7 and case 9, initially tested negative and later tested positive 9 and 26 days later, respectively (Table 3).

During hospitalization, multiple SARS-CoV-2 tests were performed per neonate to evaluate the possibility of persistent infection or the persistence of viral RNA. The range of tests per neonate was between 3 and 9, with multiple consecutive positive results supporting the presence of persistent viral RNA shedding and a possible persistent infection (Table 3).

In this cohort, only two neonates, case 2 and case 10, tested positive within the first 24 h after delivery. Two additional neonates, case 4 and case 8, tested positive between 24 and 48 h after birth. The remaining six neonates tested positive beyond the 48 h mark (Table 3).

Maternal testing showed varied timing and results. Among the mothers tested before or during labor, three were positive and two were negative. Of those tested after delivery, five tested positive and one tested negative. One mother underwent testing both before and after delivery, initially showing a negative result followed by a positive one (Table 3).

Among the neonates born to mothers with confirmed SARS-CoV-2 infection prior to delivery, two tested positive immediately at birth, which is suggestive of probable in utero transmission. In the case of the twins (case 4 and case 8), both the mother and neonates tested positive within 24–48 h after delivery, but due to the absence of maternal and neonatal testing on the exact day of birth, the route of transmission remains indeterminate. The remaining neonates were classified as early postnatal transmission, with five being possible cases and one being confirmed based on available clinical data (Table 3).

## 4. Discussion

In this case series, we analyzed data from ten newborns born between November 2020 and January 2021 who tested positive for SARS-CoV-2 at various points during their hospital stay, ranging from the first hours after birth to weeks postpartum. All neonates demonstrated adequate extrauterine adaptation, as reflected by the APGAR score. Nevertheless, six of the ten neonates died due to complications, potentially exacerbated by systemic inflammation and immunological immaturity, besides SARS-CoV-2 infection.

The high mortality rate observed in this series may not be generalized to all neonates with persistent SARS-CoV-2 infection or viral RNA positivity. As a tertiary referral center, our institution manages a high proportion of pregnancies with significant risk factors, resulting in elevated rates of prematurity, cesarean delivery, and neonatal mortality. While the national neonatal mortality rate in 2020 was 8.4 per 1000 live births [15], in contrast, our institute reported a mortality rate of approximately 30 per 1000 live births in the same year [16], of which 26% had a confirmed positive SARS-CoV-2 test. A multicentric study with a larger sample size will be necessary to allow broader generalization.

Mortality was higher among preterm neonates, accounting for five of the six deaths. Current evidence suggests that in utero transmission, particularly in mothers with moderate to severe SARS-CoV-2, increases the risk of preterm birth [10]. However, this association was not clearly observed in our cohort. Only one preterm neonate had indeterminate in utero transmission, while the neonate with possible in utero transmission was born at term.

The remaining neonates who died were classified as having possible early postnatal transmission. Preterm neonates, especially those with VLBW and ELBW, are particularly vulnerable to infections due to their underdeveloped innate and adaptive immune system. This predisposition results in longer hospitalization and an increased risk of nosocomial infection and other complications [17,18]. Notably, the two VLBW neonates in our cohort were twins that died. While not all deceased neonates were VLBW, the other two had LBW, which also increases susceptibility to infection. Large cohort studies in very-low-birth-weight infants have shown that SGA status independently increases the risk of late-onset sepsis. This increased risk has been partly attributed to altered immune maturation in growth-restricted neonates [19]. Similarly, in low-birth-weight populations, SGA status and a lower birth weight have been identified as independent risk factors for neonatal pneumonia, with SGA neonates representing a particularly vulnerable subgroup [20]. Although our case series is not designed to establish causality, the fact that two of the neonates with superinfections were classified as SGA supports the hypothesis that fetal growth restriction may contribute to immune vulnerability and adverse infectious outcomes in neonates with SARS-CoV-2 infection. These findings reinforce the need for heightened surveillance and preventive strategies in SGA neonates, particularly when additional risk factors such as prematurity and persistent viral RNA shedding are present.

Birth weight has been widely studied during the pandemic as both a risk factor and outcome. While some reports have shown a decrease in LBW during the pandemic, possibly due to lifestyle or environmental changes, LBW remains associated with an increased risk of infection in neonates [17,21]. In our cohort, eight out of ten neonates had LBW and VLBW, as shown in Table 2. The only two neonates with ABW survived without further complications, suggesting a potential protective effect.

All neonates in our study receive breast milk as their primary nutrition source, either directly or via donor milk. While breastfeeding has been associated with a reduced risk of infection and improved outcomes, particularly in VLBW neonates, we found no apparent differences in outcomes related to breastfeeding in our sample [22].

During hospitalization, neonates were monitored clinically and through laboratory testing to identify changes that could guide personalized care, including ventilatory support as needed. Among the six deceased neonates, elevated levels of C-reactive protein (CRP) and D- dimer were observed. CRP is known as a pro-inflammatory protein that plays a key role in pathogen recognition and clearance. It has been identified as a marker of disease severity and mortality in adults with SARS-CoV-2 infection [23]. Although limited data exist for neonates, our findings suggest a similar trend, even though they should be interpreted with caution given the small sample size. Previous studies have shown that CRP levels above 10 mg/L may indicate bacterial or viral sepsis, but transient elevation also occurs in uncomplicated respiratory distress or after birth stress [24]. Moreover, strengthening our finding, D-dimer has been reported to increase in neonatal sepsis and in inflammatory states associated with viral infections [25,26]. In the case of our study, these high levels were taken more than 2 weeks after birth, not showing a direct correlation with after-birth stress or uncomplicated respiratory distress, strengthening our finding, although these values should always be interpreted in conjunction with a clinical course and not as isolated predictors of severity.

While our study did not focus specifically on neonatal sepsis, we observed patterns consistent with it, as three neonates acquired a superinfection during their hospital stay. These three cases were recorded as late sepsis or urosepsis. In our cohort, four out of the six deceased neonates had leukopenia and three out of six showed thrombocytopenia. These findings align with prior evidence that show the same pattern as in past studies that lower levels of leukocytes and platelets are highly indicative of clinical neonatal sepsis [3,5,27], even without identification of the microbiological agent. This observation is noteworthy, given that the etiological agent was identified in only three cases and that all neonates had a sepsis-related cause of death.

Notably, among these neonates with superinfections, two had a very low birth weight (VLBW) and one had a low birth weight (LBW); additionally, two were classified as small for gestational age (SGA), suggesting that impaired fetal growth and reduced weight, in addition to prematurity and previous SARS-CoV-2 infection, may further increase susceptibility to secondary infections. In fungal superinfection, the synergy between Toll-like receptors (TLR) activated by SARS-CoV-2 and the nucleotide-binding oligomerization domain-like receptors (NLR) activated by *Candida* spp. may promote excessive cytokine release, contributing to hyperinflammation and a possible cytokine storm syndrome [28]. Regarding bacterial superinfection, the risk of severe complications increases significantly. Reports indicate that secondary bacterial infections contribute to a 56.7% mortality rate in affected patients, and *Klebsiella pneumoniae* is responsible for 29% of such deaths. The presence of multiple virulence factors, such as capsule production, multidrug-resistant bacteria, and biofilm production, poses a major challenge for neonates with underdeveloped immune defenses [29]. The overlap between superinfection, low birth weight, and SGA status in our cohort highlights a subgroup of particularly vulnerable neonates, underscoring the urgency of developing targeted strategies to prevent and manage secondary infections in pediatric patients with SARS-CoV-2.

Previous studies have shown an increase in coinfection and superinfection following SARS-CoV-2 acquisition. Musuuza et al. reported that up to 19% of patients with SARS-CoV-2 developed a coinfection and 24% a superinfection. These infections not only worsened the patients’ clinical conditions but were also associated with poor outcomes, including a higher incidence of mortality [30]. Feldman et al. found that such coinfections and superinfections are common in these scenarios and are even more frequent in pediatric patients, reaching up to 50%. In their review, they also concluded that these infections lead to poor outcomes, such as high mortality [31].

Another relevant finding was the persistence of positive SARS-CoV-2 results throughout hospitalization in all neonates. This suggests that there is a prolonged pro-inflammatory state, which may interfere with proper recovery. Such a sustained immune activation increases the risk of adverse outcomes such as systemic inflammatory response syndrome (SIRS), sepsis, and death. The immunological vulnerability of neonates provides a plausible explanation for these findings, and the current lack of standardized treatment guidelines emphasizes a critical gap in care that must be addressed [32,33].

Finally, we examined weight gain during hospitalization. As expected, critically ill neonates who died showed inadequate weight gain from birth until death, whereas among survivors, two exhibited excessive weight gain and two insufficient weight gain. Although poor weight gain is a known marker of illness severity, limited data exist regarding its relationship with SARS-CoV-2-associated infections in neonates. It is plausible that infection-induced inflammation contributes to a catabolic state, which may compromise energy reserves and immune function [34]. However, this hypothesis requires confirmation in larger cohorts, and our findings should be interpreted as exploratory rather than causal

The high mortality rate observed in our case series reflects the interplay of multiple risk factors, including prematurity, low birth weight, persistent SARS-CoV-2 positivity during hospitalization, the presence of bacterial and fungal superinfections, and a strong inflammatory response indicated by elevated CRP and D-dimer levels. Among the six deceased neonates, three were classified as SGA and three as AGA, indicating that adverse outcomes were not limited to growth-restricted infants but also affected neonates with adequate fetal growth. Previous studies have demonstrated that prematurity, LBW, and incidence of SGA are major predictors of morbidity and mortality, and when combined with SARS-CoV-2 infection, they further increase the risk of death [9,18,35]. At our institute, Cardona-Perez et al. (2021) reported a 28% SARS-CoV-2 positivity rate among 250 pregnant women, with 23% of their neonates testing positive and 44% of them being preterm [36]. Similarly, Hernández-Cruz et al. (2021) found a 30.7% positivity rate in pregnant women during a one-year screening period [37], markedly higher than the global prevalence of 9% reported in a 2025 meta-analysis of 293,152 pregnancies (95% CI: 7–10%), which also identified higher risks of NICU admission and neonatal death (OR 2.35; 95% CI: 1.16–4.76). Consistently, UK data from 2019 to 2021 indicated that 4.8% of neonatal deaths occurred among infants born to SARS-CoV-2-positive mothers, with prematurity remaining a major contributing factor [38].

## 5. Conclusions

In conclusion, this case series reflects the complex clinical course that SARS-CoV-2 can trigger in neonates, shown by persistent qPCR positivity, pro-inflammatory states, and high rates of adverse outcomes. Despite having correct adaptation at birth and nutritional support, several neonates develop complications such as sepsis, superinfections, inadequate weight gain, and ultimately death. The findings suggest that factors like prematurity, low birth weight, and early postnatal infection may contribute significantly to poor prognosis.

The clinical relevance of these findings lies in highlighting the need for heightened surveillance and individualized management strategies in Neonatal Intensive Care Units. Persistent viral positivity and prolonged inflammatory responses may warrant closer laboratory monitoring, early identification of secondary infections, and personalized supportive care, particularly in preterm and low-birth-weight neonates. These observations support the importance of interdisciplinary care and reinforce the need to optimize infection control measures, nutritional strategies, and close follow-up in neonates with confirmed SARS-CoV-2 infection. Larger, prospective, and multicenter studies are needed to better define transmission pathways, identify prognostic factors, and establish SARS-CoV-2 infection. Addressing these gaps will be essential to improve outcomes and ensure compressive and effective care for this highly vulnerable population.

## Figures and Tables

**Table 1:** Maternal clinical characteristics and outcomes.

Variable	Case 1	Case 2	Case 3	Case 4	Case 5	Case 6	Case 7	Case 8	Case 9	Case 10
Maternal Age (years)	19	26	37	25	22	22	34	25	29	25
No. of Pregnancies	1	2	2	1	1	1	2	1	1	2
History of Abortions	0	0	0	0	0	0	0	0	0	1
History of Cesarean	0	1	1	0	0	0	1	0	0	0
Diabetes	No	No	Yes	No	No	No	Yes	No	No	No
Hypertension	No	No	No	No	No	No	No	No	No	No
Other Diseases *	Yes **	No	Yes ***	No	No	No	No	No	No	No
Preterm Birth (present)	Yes	No	Yes	Yes	Yes	Yes	No	Yes	Yes	No
Type of Pregnancy Resolution (present)	Cesarean	Cesarean	Cesarean	Cesarean	Cesarean	Cesarean	Cesarean	Cesarean	Cesarean	Cesarean
Obstetric Hemorrhage (present)	No	No	Yes	No	No	No	No	No	No	Yes
Maternal SARS-CoV-2 Result at Birth	Negative	Positive	NT	Positive	NT	NT	Negative	Positive	Positive	Positive

No.—number; present—current birth; NT—not tested; and gray shading indicates cases of neonatal death. * Various diseases were searched and analyzed in these women, searching for immunological diseases, cardiovascular diseases, and respiratory diseases, among others. ** Suffered from epilepsy, which was ruled out as a risk factor or confounder, being irrelevant for this case series analysis. *** Severe preeclampsia: This was the only case classified as an iatrogenic preterm delivery. Cases resulting in death are highlighted with a gray background.

**Table 2:** Neonatal clinical characteristics and outcomes.

Variable	Case 1	Case 2	Case 3	Case 4	Case 5	Case 6	Case 7	Case 8	Case 9	Case 10
Gestational Age (weeks)	33.4	37.5	33.4	33.6	31	31	39.3	33.6	34.2	37.5
Sex	Male	Male	Male	Female	Male	Male	Female	Female	Female	Male
Antenatal Corticosteroids Therapy	Yes	No	No	No	Yes	Yes	No	No	Yes	No
Apgar 1	6	7	8	8	5	3	8	2	7	8
Apgar 5	8	9	9	9	8	8	9	9	8	9
Birth Weight (g)	1865	2335	1570	1725	1105	1235	3155	1515	1885	3635
Birth Length (cm)	44.5	46.5	40	41	37	37	49	40	44	49
Birth Weight Classification	LBW	LBW	LBW	LBW	VLBW	VLBW	ABW	LBW	LBW	ABW
Fenton Growth- Percentile	24	4	8	18	9	16	37	7	22	88
Fenton Growth- Classification	AGA	SGA	SGA	AGA	SGA	AGA	AGA	SGA	AGA	AGA
D-Dimer	High	High	Normal	Normal	High	Normal	Normal	Normal	Normal	Normal
Superinfection	Yes *	Yes *	Yes *	No	No	No	No	No	No	No
Corticosteroid Treatment for	No	No	No	No	No	No	No	No	No	No
Hospital Stay (days)	51	118	54	42	43	43	69	20	57	18
Discharge Weight (g)	2420	3590	2675	2256	1890	1915	5048	1980	2710	3550
Discharge Length (cm)	44.5	56	41	45	39	37	51	41	47	49
Real Weight Gain (g) **	555	1255	1105	531	785	680	1893	465	825	−85
Weight Gain Classification	Inadequate	Inadequate	Inadequate	Inadequate	Inadequate	Inadequate	Excessive	Excessive	Inadequate	Inadequate
Cause of Death	Septic Shock, SARS-CoV-2, and Prematurity	Respiratory Failure, Late Sepsis	Septic Shock, SARS-CoV-2, and Prematurity	Septic Shock, Late Sepsis	Cardio-respiratory Arrest, Septic Shock, and Prematurity	Septic Shock, Respiratory Distress	NA	NA	NA	NA

Apgar 1—Apgar score assessed 1 min after birth; Apgar 5—Apgar score assessed 5 min after birth; g—gram; cm—centimeter; LBW—low birth weight; VLBW—very low birth weight; ABW—adequate birth weight; SGA—small for gestational age; AGA—appropriate for gestational age; and NA—not applicable. * Case 1 was superinfected with *Candida parapsilosis*, case 3 was superinfected by *Klebsiella pneumoniae*, and case 4 was superinfected with *Candida albicans*. The three microorganisms were detected in blood or urine cultures, and the cases were classified as sepsis or urosepsis. ** Real weight gain was calculated taking into consideration the initial drop in newborn weight during the first 10–14 days. Cases resulting in death are highlighted with a gray background.

**Table 3:** SARS-CoV-2 characterization in infected neonates.

Case	Preterm Birth	No. of SARS-CoV-2 Tests	Positive SARS-CoV-2 Tests	Positive SARS-CoV-2 at Age <24 h	Positive SARS-CoV-2 at Age 24–48 h	Positive SARS-CoV-2 at Age >48 h	SARS-CoV-2 Test Before/At Delivery (Mother)	SARS-CoV-2 Test After Delivery (Mother)	Transmission Classification	Evidence for Transmission Classification
Case 1	Yes	7	6/7	NT	NT	Yes	Negative	NT	Possible Early Postnatal Transmission	Evidence includes a positive RT-PCR result from a nasal/saliva swab collected after 48 h of life, followed by another positive test 10 days later, with no positive results from sterile samples within the first 48 h. The mother tested negative at delivery.
Case 2	No	8	6/8	Yes	Yes	Yes	Positive	NT	Possible In Utero Transmission	Evidence includes a positive RT-PCR result from a nasal/saliva swab collected at birth (within the first 24 h of life), followed by a second positive test from the same sample type between 24 and 48 h. The mother tested positive by nasopharyngeal swab prior to delivery.
Case 3	Yes	8	5/8	NT	NT	Yes	NT	Negative	Possible Early Postnatal Transmission	Evidence includes a positive RT-PCR result from a nasal/saliva swab collected after 48 h of life, followed by another positive test 10 days later, with no positive results from sterile samples within the first 48 h. The mother’s SARS-CoV-2 status was unknown.
Case 4	Yes	7	5/7	NT	Yes	Yes	NT	Positive	Indeterminate In Utero Transmission	Evidence includes a positive RT-PCR result from a nasal/saliva swab collected between 24 and 48 h followed by a second positive test from the same sample type after 48 h. The mother tested positive by nasopharyngeal swab after delivery.
Case 5	Yes	7	4/7	NT	NT	Yes	NT	Positive	Possible Early Postnatal Transmission	Evidence includes a positive RT-PCR result from a nasal/saliva swab collected after 48 h of life, followed by another positive test 10 days later, with no positive results from sterile samples within the first 48 h. The mother’s SARS-CoV-2 status at delivery was unknown, but she tested positive after delivery.
Case 6	Yes	6	4/6	NT	NT	Yes	NT	Positive	Possible Early Postnatal Transmission	Evidence includes a positive RT-PCR result from a nasal/saliva swab collected after 48 h of life, followed by a subsequent positive test 10 days later, with no positive results from sterile samples within the first 48 h. The mother’s SARS-CoV-2 status at delivery was unknown, but she tested positive after delivery.
Case 7	No	9	6/9	NT	No	Yes	Negative	Positive	Possible Early Postnatal Transmission	The neonate tested negative between 24 and 48 h after birth but had a positive RT-PCR result from a nasal/saliva swab after 48 h of life, followed by another positive test 10 days later. No positive results were detected from sterile samples within the first 48 h. The mother tested negative at delivery but was positive after delivery.
Case 8	Yes	3	3/3	NT	Yes	Yes	NT	Positive	Indeterminate In Utero Transmission	Evidence includes a positive RT-PCR result from a nasal/saliva swab collected between 24 and 48 h of life, with the mother testing positive after delivery.
Case 9	Yes	7	4/7	No	No	Yes	Positive	NT	Confirmed Early Postnatal Transmission	Evidence includes a positive RT-PCR result from a nasal or saliva swab collected after 48 h of life, followed by another positive test 10 days later, with the mother testing positive at delivery.
Case 10	No	4	3/4	Yes	Yes	Yes	Positive	NT	Possible In Utero Transmission	Evidence includes a positive RT-PCR result from a nasal or saliva swab collected within the first 24 h of life, followed by a second positive test from the same sample type between 24 and 48 h, with no evidence of positivity in sterile neonatal samples. The mother tested positive by nasopharyngeal swab at delivery.

Clinical data from 10 patients, including SARS-CoV-2 test results, maternal infection status before and at birth, and patient outcomes (alive or deceased). It also indicates the number of tests performed, the proportion of positive results, and whether the birth was preterm. This analysis explores the potential link between maternal SARS-CoV-2 infection and neonatal outcomes. No.—number; h—hours; mother—maternal data; NT—not tested. Cases resulting in death are highlighted with a gray background.

## Data Availability

The original contributions presented in this study are included in the article. Further inquiries can be directed to the corresponding author.
